# Targeting Oncostatin M Receptor to Attenuate Carotid Artery Plaque Vulnerability in Hypercholesterolemic Microswine

**DOI:** 10.26502/fccm.92920380

**Published:** 2024-05-08

**Authors:** Jerry Trinh, Jennifer Shin, Vikrant Rai, Devendra K Agrawal

**Affiliations:** Department of Translational Research, Western University of Health Sciences, Pomona, California 91763, USA

**Keywords:** Atherosclerosis, Carotid stenosis, CDK inhibition, Inflammation, Plaque vulnerability, Oncostatin M

## Abstract

Atherosclerosis is a chronic inflammatory disease that leads to acute embolism via the formation of atherosclerotic plaques. Plaque formation is first induced by fatty deposition along the arterial intima. Inflammation, bacterial infection, and the released endotoxins can lead to dysfunction and phenotypic changes of vascular smooth muscle cells (VSMCs), advancing the plaque from stable to unstable form and prone to rupture. Stable plaques are characterized by increased VSMCs and less inflammation while vulnerable plaques develop due to chronic inflammation and less VSMCs. Oncostatin M (OSM), an inflammatory cytokine, plays a role in endothelial cells and VSMC proliferation. This effect of OSM could be modulated by p27^KIP1^, a cyclin-dependent kinase (CDK) inhibitor. However, the role of OSM in plaque vulnerability has not been investigated. To better understand the role of OSM and its downstream signaling including p27^KIP1^ in plaque vulnerability, we characterized the previously collected carotid arteries from hyperlipidemic Yucatan microswine using hematoxylin and eosin stain, Movat Pentachrome stain, and gene and protein expression of OSM and p27^KIP1^ using immunostaining and real-time polymerase chain reaction. OSM and p27^KIP1^ expression in carotid arteries with angioplasty and treatment with either scrambled peptide or LR12, an inhibitor of triggering receptor expressed on myeloid cell (TREM)-1, were compared between the experimental groups and with contralateral carotid artery. The results of this study elucidated the presence of OSM and p27^KIP1^ in carotid arteries with plaque and their association with arterial plaque and vulnerability. The findings suggest that targeting OSM and p27^KIP1^ axis regulating VSMC proliferation may have therapeutic significance to stabilize plaque.

## Introduction

Atherosclerosis, a chronic inflammatory disease of the vasculature, is a major global health problem, leading to many acute and chronic cardiovascular diseases such as heart attacks, and strokes. Atherosclerosis is characterized by thickening and hardening of the arteries caused by a buildup of plaques along the intima layers of arteries. Stable plaques are characterized by thick collagen-rich fibrous caps and lipid-rich mass formed from the oxidation of deposited lipids called minimally oxidized lipids (oxLDL) [1,2]. Fat accumulation in the form of foam cells inside the plaque has been associated with plaque vulnerability, activating both the innate and adaptive immune system and increasing inflammation and inflammatory cytokines. While stable plaques are dense with collagen and vascular smooth muscle cells (VSMCs), unstable plaques are rich in macrophages and poor in collagen or VSMCs [3]. Atherosclerotic plaque stability is determined by the balance between degradation and formation of the extracellular matrix (ECM) [3]. Similarly, LPS, a bacterial endotoxin, and other inflammatory mediators render plaque vulnerable and increase the risk of ischemic events. ECM degradation can occur by matrix metalloproteinase (MMP), an enzyme secreted by activated macrophages and T-lymphocytes present in atherosclerotic plaques. Increased expression of inflammatory cells, damage-associated molecular patterns (DAMPs) [4,5], mediators of inflammation including triggering receptor expressed on myeloid cells (TREM-1) on macrophages and dendritic cells [4], and induction of TREM-1 with TNF-α [3] increase local inflammation and plaque vulnerability. Thus, attenuating inflammation by targeting these mediators has been proposed to stabilize plaque and may also reduce plaque burden. Degradation of collagen also weakens the fibrous cap, a structural support of the plaque, and leaves the plaque vulnerable to rupture [6]. In addition, inflammation renders the stable plaque unstable resulting in thrombus formation and ischemic events [3–5]. Excessive proliferation and migration of VSMCs play a role in the pathobiology of atherosclerosis [7]. Atherosclerosis progresses by enlarging and thickening the elastic and muscular arteries [8]. Thus, understanding the molecular mechanisms that control hyperplastic growth and the migration of VSMCs will aid in the development of novel strategies to reduce neointimal thickening [7].

Oncostatin M (OSM) is a member of the interleukin (IL)-6 related cytokine secreted by immune cells mainly macrophages, neutrophils, and T cells [9]. OSM is also involved in inflammation, oxidative stress, VSMC proliferation, migration, differentiation, survival, angiogenesis, and ECM remodeling [10,11]. These molecular events play critical role in plaque formation, progression, and rupture. OSM exerts a proinflammatory effect on endothelial cells, stimulates VSMC proliferation, and ultimately leads to neointimal hyperplasia (NIH) [12]. p27^KIP1^, a member of the Kip family of cyclin-dependent kinase (CDK) inhibitors, inhibits cellular change that normally occurs during cell locomotion [13]. In particular, suppression of p27^KIP1^ has been associated with the suppression of smooth muscle cell proliferation and vascular remodeling in the pulmonary artery smooth muscle cells of mouse model [13] and in cultured cells [14]. This study was designed to investigate the expression of OSM and p27^KIP1^ in carotid arteries with plaque and following treatment with anti-inflammatory agent LR12 inhibiting TREM-1 which is a mediator of inflammation and promoter of plaque vulnerability [5,15]. The association of OSM and p27^KIP1^ expression with VSMC proliferation and plaque vulnerability was analyzed. The results suggest that differential expression of OSM and p27^KIP1^ is associated with plaque formation and vulnerability and targeting OSM- p27^KIP1^ axis may have therapeutic significance in attenuating plaque vulnerability.

## Material and Methods

### Tissue collection and processing

This study used the previously collected carotid arterial tissues from hyperlipidemic Yucatan microswine (protocol # R19IACUC026, approved by Western University IACUC). Tissue samples were collected from both the right internal carotid artery (RIC) undergoing angioplasty and treatment with LR12 and scrambled peptide and left internal carotid artery (LIC) without any intervention. The tissue was processed using a tissue processor and a 5μm thin sections were used in all experiments. Tissues from a minimum of 7 swine in each group (8 LR12 treated group, 9 scrambled peptide treated group, and 7 LICs) were sampled for the experiments. All RIC samples were exposed to ox-LDL after intimal injury by angioplasty to induce plaque formation. We used a power analysis with an α value of 0.05. The sample size necessary to have at least 90% power to detect significant change is 7 in each group.

### Hematoxylin and Eosin and Pentachrome staining

Hematoxylin and eosin (H&E) staining was done following the standard protocol in our lab. Briefly, after deparaffinization and rehydration of the slides through a series of xylene, ethanol, and distilled water, the tissue sections were stained with hematoxylin (45 seconds) followed by eosin (8–10 dips). The stained slides were mounted with xylene-based mounting media. Movat-Pentachrome staining was done using a modified pentachrome kit (Pentachrome Stain Kit - Russell Movat, SKU # KTRMPPT EA) following the manufacturer’s protocol and standard lab procedure. Stained tissue sections were scanned at 100μm using a light microscope (Leica DM6). All the scanned images were blindly reviewed by at least one independent observer. Three-to-four images from each stained section and three sections from each tissue were scanned for evaluation. H&E images were evaluated for plaque size, intima-media thickness, inflammation, and plaque rupture while Movat-Pentachrome stained slides were used to evaluate ECM remodeling.

### Immunostaining

Immunohistochemistry was performed using the peroxidase anti-peroxidase method using a secondary antibody conjugated to horseradish peroxidase. The paraffin fixed sections were deparaffinized, rehydrated, and antigen retrieved using 1% citrate buffer (Sigma Aldrich # C9999) before immunostaining as per the standard protocol in our laboratory. Briefly, the slides were washed with phosphate-buffered saline (PBS) after blocking, and tissue was encircled using Pep Pen. The tissue samples were incubated with 3% hydrogen peroxide (Sigma Aldrich # H1009) for 15 minutes and washed with PBS for 5 minutes each three times. Blocking of non-specific binding was done using the blocking solution from Vectastain Elite ABC kit (Vector Labs) and the tissues were incubated for 1 hour at room temperature. After tipping off the blocking solution, the tissue sections were incubated overnight at 4°C with the primary antibodies (OSM, ab198830; mTOR, Cell Signaling 2971; p27^kip1^, ab193379; α-SMA, ab7817; PCNA, ab29). The slides were washed 3 times 5 minutes each with 1x PBS and then incubated with the secondary antibody from the Vectastain Elite ABC kit for 1 hour at room temp. The slides were rinsed 3 times with 1x PBS, followed by incubation with the Vectastain ABC horseradish peroxidase (HRP) for 30 minutes at room temp. The tissue sections were then rinsed with 1x PBS followed by incubation with 3,3′-diaminobenzidine (DAB) (Thermo Scientific, Cat # 34002) or AEC (3-amino-9-ethylcarbazole) HRP substrate for 2 to 5 minutes until the development of brown/red color. Tissue sections were washed with water once and then stained with hematoxylin for 5–10 seconds. The slides were rinsed in running tap water for 5 minutes and mounted with a xylene-based mounting medium. The stained slides were imaged with a Leica DM6 microscope at a scale of 100 μm. The high-magnification images from each tissue section were analyzed using ImageJ for average stained intensity and average stained area. Three sections for each tissue and three-to-four images from each tissue were used for evaluation and statistical analyses.

### Quantitative Real-Time Polymerase Chain Reaction

Total RNA was extracted using TRIZOL reagent (T9424, Sigma, St Louis, MO, USA) following the manufacturer’s instructions and RNA yield was measured using Nanodrop 2000. The cDNA was prepared using an iScript kit (#1708891, BioRad, USA) following the manufacturer’s instructions. Real-time PCR (RT-PCR) was performed in triplicate using SYBR Green with CFX96 RT-PCR system (BioRad Laboratories, Hercules, CA, USA). The primers (Table 1) were obtained from Integrated DNA Technologies (Coralville, IA, USA). The PCR cycling conditions were 5 min at 95°C for initial denaturation, 40 cycles of 30 s each at 95°C (denaturation), 30 s at 55–60°C (according to the primer annealing temperatures), and 30s at 72°C (extension) followed by melting curve analysis. Fold-change in mRNA expression relative to controls was analyzed using the 2^-^^CT^ method after normalization with 18S. Each experiment was repeated for three biological replicates (n=3). RT-PCR was performed to evaluate the mRNA expression of OSM, OSMR, Ki-67, alpha-smooth muscle actin (αSMA), and housekeeping gene 18S. Ki-67 and αSMA served as markers for VSMC proliferation. Sample groups included 7 LIC tissues treated with LR12, 8 LIC tissues treated with scrambled peptide, 8 RIC tissues treated with LR12, and 7 RIC tissues treated with scrambled peptide.

### Statistical analysis

Data were analyzed using GraphPad Prism 9 and presented as the mean ± SEM. The comparison between two groups for the expression of the protein of interest was performed using Student’s t-test and between more than two groups using One-way ANOVA with Bonferroni’s post-hoc correction. A probability (*p*) value of < 0.05 was accepted as statistically significant.

## Results

### Histological analysis

**Figure 1:**
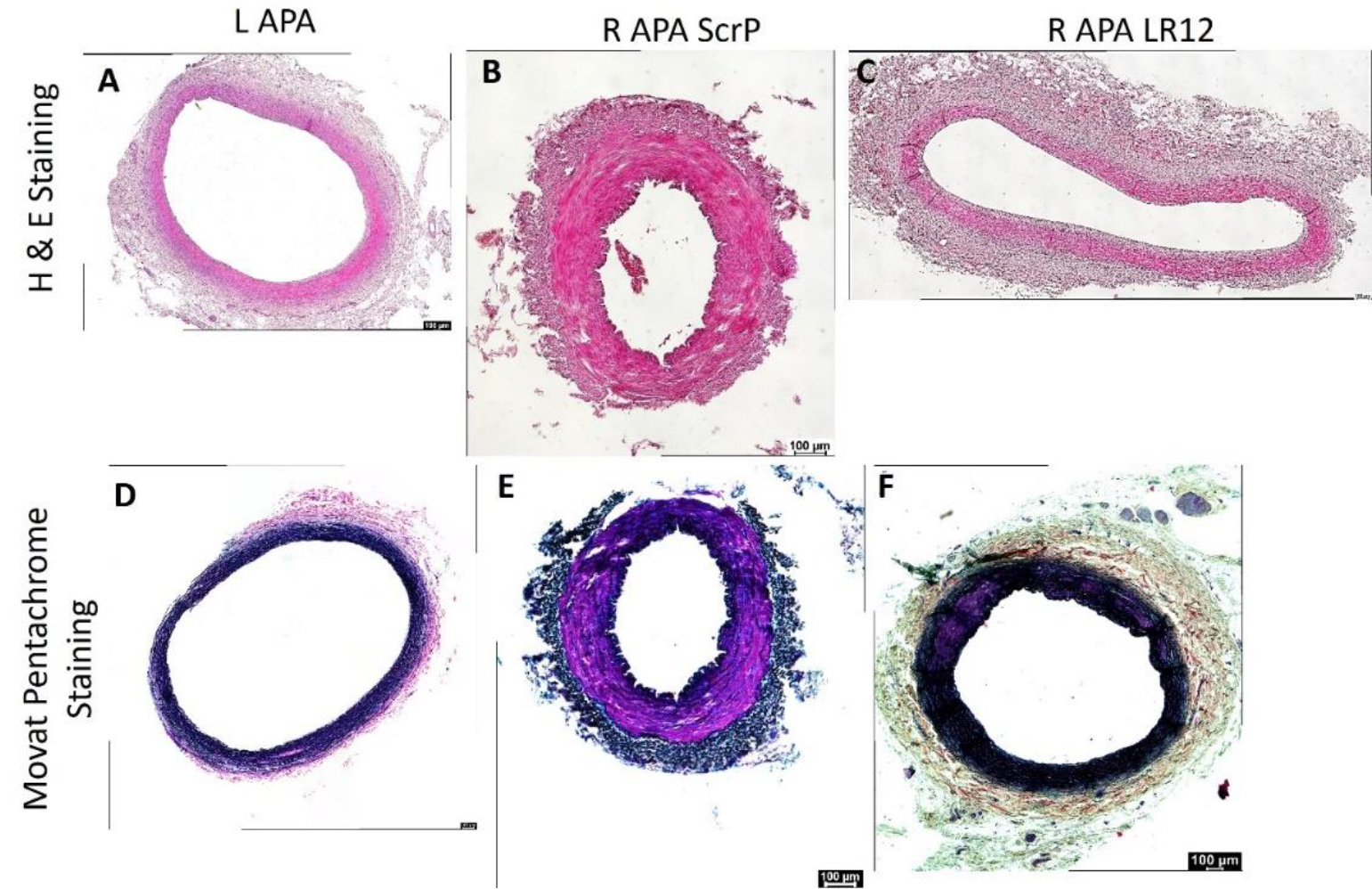
H&E and Movat Pentachrome staining in left and right ascending pharyngeal arteries. These are representative images of all swine arteries included in this study. H&E staining (panels A-C) and Movat Pentachrome staining (panels D-F).

H&E staining at the site of intervention (right ascending pharyngeal artery, RAPA) and treated with scrambled peptide revealed neointimal hyperplasia, presence of plaque, media thickening, and lumen stenosis while arteries treated with LR12 were of normal histology except one artery with minimal neointimal thickening on 1/3^rd^ arterial intima. The left APA showed normal histology. RAPA showed diffuse medial inflammatory cell infiltration in the scrambled treated group while arteries in the LR12 peptide treatment showed minimal to no inflammation in the medial layer. The contralateral LAPA also showed mild to minimal inflammation in the medial layer of the arteries (Figure 1 panels A-C). Movat-Pentachrome analysis revealed increased purple and black stain suggestive of elastin staining in arteries treated with scrambled peptide and LR12 peptide while the staining in left APAs was normal (Figure 1 panels D-F). The elastin staining pattern was not clear and was suggestive of elastin degradation in the scrambled peptide and LR12 peptide treated groups.

### Real-time polymerase chain reaction

#### Treatment with ox-LDL and LR12 peptide significantly decreased expression of OSM and OSMR in injured carotid arteries

**Figure 2:**
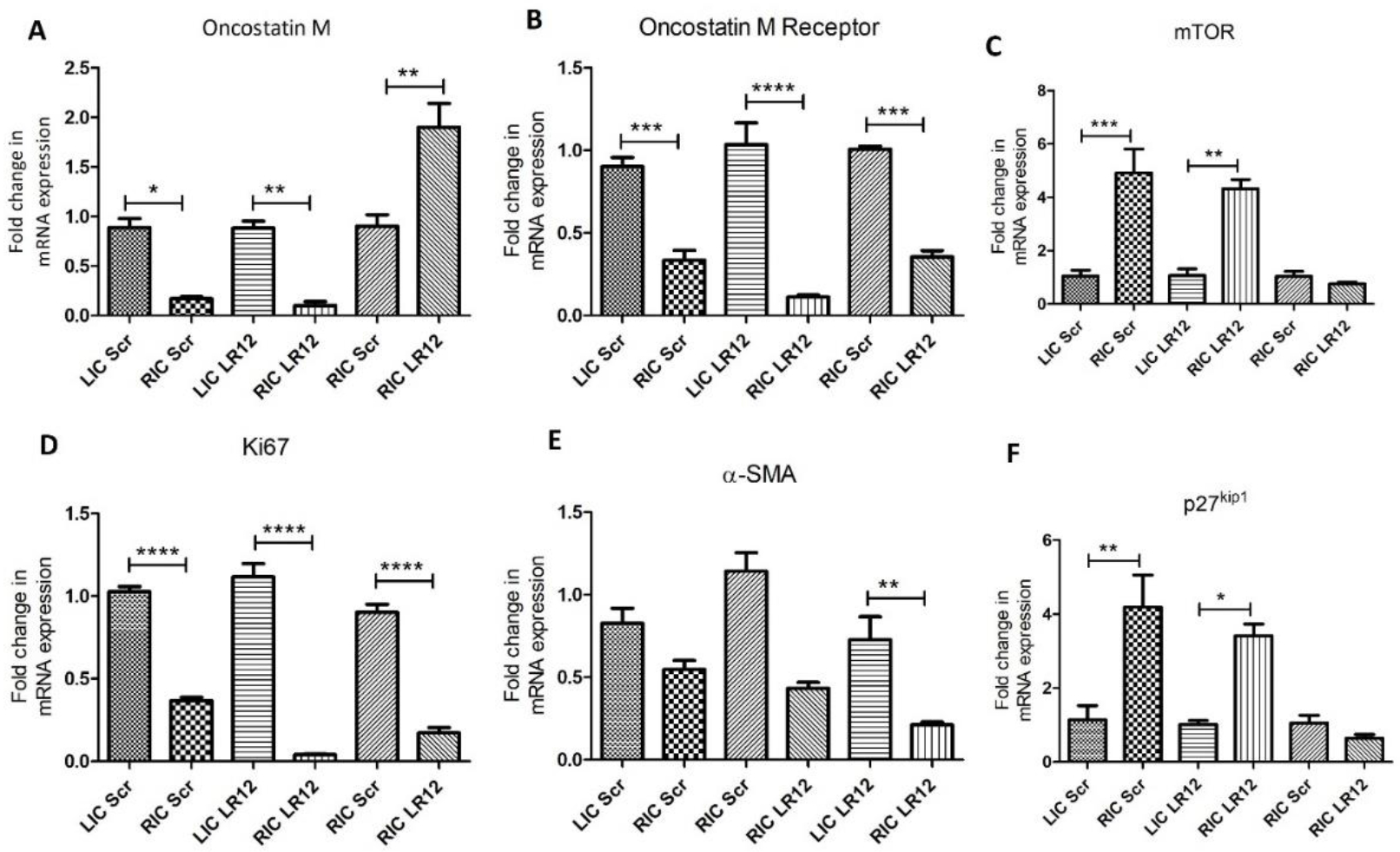
Real-Time Polymerase Chain Reaction to evaluate mRNA transcripts. OSM- oncostatin M (panel A), OSMR-oncostatin M receptor (panel B), mechanistic Target of Rapamycin (mTOR; panel C), α-SMA- Ki67- cell proliferation marker (panel D), alpha-smooth muscle actin (panel E), and p27kip1 (panel F). Data are presented as the mean ± SEM. *p<0.05, **p<0.01, ***p<0.001,and ****p<0.0001.

Tissues from RAPA (equivalent to RIC) showed significantly decreased expression of OSM in comparison to tissues from the LAPA in scrambled peptide treated group (p = 0.0179) and LR12 peptide treated group (p = 0.00598) (Figure 2 panel A). Further, compared to scrambled peptide-treated RAPA, OSM expression was significantly increased in LR12-treated RAPA (p = 0.01). RT-PCR analysis for OSMR revealed significantly decreased mRNA expression in RAPA in comparison to the LAPA artery treated with LR12 (0.00506) and with scrambled peptide (p=0.00232). There was significantly decreased expression of OSMR in RAPA arteries treated with LR12 peptide compared to arteries treated with scrambled peptide (p = 0.00017) (Figure 2 panel B).

#### Treatment with ox-LDL and LR12 peptide decreased expression of Ki67 and mTOR compared to the treatment with scrambled peptide

RT-PCR analysis for mTOR revealed significantly increased mRNA expression in RAPA in comparison to the LAPA artery treated with LR12 (0.00157) and with scrambled peptide (p=0.0132). There was decreased expression of mTOR in RAPA arteries treated with LR12 peptide compared to arteries treated with scrambled peptide but was not statistically significant (Figure 2 panel B). RT-PCR analysis revealed significantly decreased expression of Ki67 in RAPA treated with LR12 compared to the contralateral artery (p= 0.000127) and in RAPA treated with scrambled peptide compared to LAPA (p= 0.0000557). Further, compared to scrambled peptide-treated RAPA, Ki-67 expression was significantly decreased in LR12-treated RAPA (p = 0.00024) (Figure 2 panel D).

#### Treatment with ox-LDL and LR12 significantly decreased expression of α-SMA and p27^kip1^ compared to treatment with scrambled peptide

RT-PCR showed significantly decreased expression of α-SMA in RAPA treated with LR12 compared to RAPA treated with scrambled peptide (p=0.0038). The mRNA expression of α-SMA was decreased in RAPA treated with LR12 and scrambled peptide compared to LAPA (Figure 2 panel E). The results also revealed decreased but not statistically significant expression of p27^kip1^ in RAPA treated with LR12 compared to RAPA treated with scrambled peptide. The mRNA expression of p27^kip1^ was significantly decreased in RAPA treated with LR12 (p=0.00218) and scrambled peptide (p=0.0328) compared to LAPA (Figure 2 panel F).

#### Immunohistochemistry

**Figure 3:**
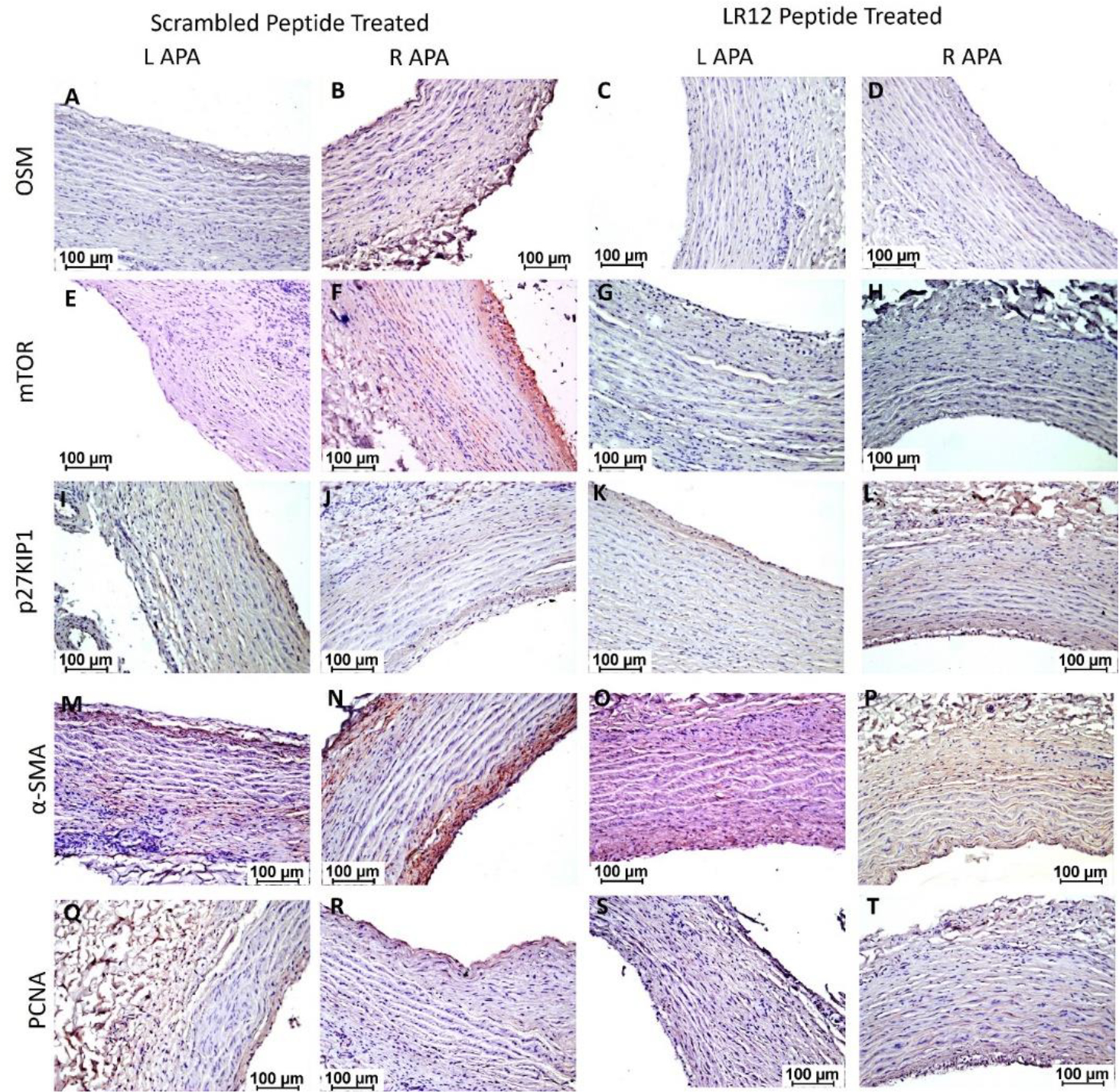
Immunohistochemistry for OSM, mTOR, p27KIP1, α-SMA, and PCNA in right and left ascending pharyngeal arteries. OSM-oncostatin M (Figure 3 panels A-D), mTOR- mammalian target of rapamycin (Figure 3 panels E-H), α-SMA-alpha smooth muscle actin (Figure 3 panels M-P), and p27KIP1 (Figure 3 panels I-L), and PCNA- proliferating cell nuclear antigen (Figure 3 panels Q-T). All images were scanned at 100μm. These are representative images from all swine included in this study.

Immunohistochemistry (IHC) revealed immunopositivity for OSM, mTOR, p27^KIP1^, α-SMA, and PCNA in ascending pharyngeal arteries (APA). The immunopositivity for OSM (Figure 3 panels A-D), mTOR (Figure 3 panels E-H), α-SMA (Figure 3 panels M-P), and PCNA (Figure 3 panels Q-T) was increased in RAPA treated with scrambled peptide compared to arteries treated with LR12 peptide and contralateral left arteries except for p27^KIP1^ (Figure 3 panels I-L) where immunopositivity was more in RAPA treated with LR-12 compared to RAPA treated with scrambled peptide and LAPA.

## Discussion

Atherosclerosis is characterized by plaque formation in the intimal layer of arteries, causing thickening and potential obstruction of these vessels. Plaques may be characterized as either stable or unstable based on the composition of the extracellular matrix [3]. While stable plaques have been characterized as primarily composed of collagen and VSMCs, unstable plaques tend to be denser in macrophages but lighter in collagen and VSMCs [3]. In particular, the proliferation and migration of VSMCs have been shown to play a role in the pathogenesis of atherosclerosis [7]. One key factor in the regulation of VSMC proliferation and migration is Oncostatin M, a cytokine of the IL-6 family secreted by innate immune cells. The proinflammatory effect of OSM on vascular endothelium activates the proliferation of VSMCs in a pathway mediated by p27^kip1^ and proliferation of pulmonary artery smooth muscle cells is associated with a decrease in expression of p27^kip1^ [13]. Further, proliferating cells have less stable p27^kip1^ expression compared to non-proliferating cells [16]. Since both OSM and p27^kip1^ regulate cell proliferation which in turn is associated with plaque vulnerability, we investigated the expression of p27^kip1^ and OSM expression and their association with VSMC proliferation in hyperlipidemic microswine carotid arteries undergone angioplasty to induce atherosclerotic plaque and treated with or without TREM-1 inhibitor, LR12 [15]. An increased presence of inflammation, neointimal formation, medial thickening, and plaque formation in RAPAs treated with scrambled peptide compared to LR12-treated arteries in this study supports the potency of ox-LDL in inducing plaque formation and inflammatory cell recruitment and the beneficial effects of LR12 peptide in attenuating inflammation and plaque formation [15,17,18]. This difference may have been associated with the effects of LR12, which has previously been shown to dampen the pro-thrombogenesis effects of TREM-1 [19]. LR12 inhibits the expression of tissue factors on monocytes and may reduce their activity in leukocyte-associated thrombosis. The decrease in artery wall diameter and stenosis of the lumen in RAPA treated with scrambled peptide in this study may indicate healing, associated with intimal hyperplasia and luminal narrowing. This process may be due to the replacement of the inflamed tissue with fibrotic remodeling which occurs over months after the initial insult [20]. This finding is supported by the increased fibrosis in arteries observed by Movat-Pentachrome staining. This may be explained by the pathogenesis of atherosclerosis, in which oxidized LDL increases the deposition of cholesterol. The intimal damage induced by cholesterol may trigger a response by smooth muscle cells to increase the production or expression of elastin, collagen, and glycan [17].

The presence of oxidized LDL triggers an inflammatory response pathway associated with VSMC proliferation and migration as well as plaque formation [17,18]. A significantly decreased mRNA expression of α-SMA in LR12 treated RAPA compared to their left counterparts and scrambled treated RAPA support the notion that oxLDL promotes inflammation and cell proliferation and thus plaque formation while attenuation of inflammation by LR12 is beneficial to decrease neointima and plaque formation, as supported by the results of this study. Chen et al. [21] reported that reduced expression of α-SMA after intimal injury is associated with an increased proliferation and migration of smooth muscle cells with a proinflammatory phenotype of VSMCs. The decreased expression of α-SMA in our tissues is supported by the findings of decreased mRNA expression of early fibrosis markers such as α-SMA and procollagen-1 in alcohol-fed mice [21]. Interestingly, in our IHC study, we found that immunopositivity for α-SMA was higher in the intimal area in the scrambled peptide-treated group suggestive of VSMCs migration from medial to intimal layer during intima and plaque formation [22] compared to a diffuse staining in the medial layer in LR12-treated RAPA and contralateral arteries suggestive of VSMCs proliferation but no migration. A significant decrease in proliferation marker Ki67 expression, a nuclear protein upregulated in the G1, S, and G2 phases of the cell cycle, in RAPA tissues treated with LR12 compared to the RAPA tissues treated with scrambled peptide suggests cell proliferation during plaque formation and the beneficial effect of LR12 in attenuating inflammation and neointimal hyperplasia. Increased VSMCs proliferation is a hallmark of plaque formation and stable plaque [22] but attenuated VSMCs in an advanced plaque prevent plaque rupture [23]. This may be further corroborated by a study in which oxLDL-treated human aortic vascular smooth muscle cells demonstrated increased Ki67 protein expression [24]. However, the findings of significantly decreased mRNA transcript in RAPA treated with LR12 or scrambled peptide compared to LAPA without intervention raises concerns and this may be due to VSMCs apoptosis in chronic atherosclerotic plaques (the tissues were collected 6 months after intimal injury), medial atrophy, elastin fragmentation, and fibrosis [23,25]. These findings are supported by a nearly similar pattern of immunopositivity of proliferating cell nuclear antigen (PCNA) in left and right arteries on IHC. The immunopositivity for PCNA was higher in RAPA (plaque and intima region) treated with scrambled peptide compared to RAPA and LAPA. p27^Kip1^ is a cyclin-dependent kinase inhibitor expressed in VSMCs. Past studies have shown that in response to serum stimulation, VSMCs demonstrate decreased expression of p27^kip1^ and increased mitogenesis due to decreased binding of p27^kip1^ to the cell cycle enzyme cyclin E [13]. This suggests that decreased expression of p27^kip1^ is associated with cell proliferation while attenuating p27^kip1^ may proliferate cells. In the context of atherosclerosis, p27^kip1^ has been a target of inhibition to stimulate VSMC proliferation while studying plaque formation. A significantly increased p27^kip1^ in RAPA treated with LR-12 and scrambled peptide compared to their left counterpart found in this study may be due to ongoing VSMCs and ECs proliferation during neointimal hyperplasia and plaque formation while decreased expression in LR1–12 treated RAPA compared to scrambled-peptide treated RAPA may be due to decreased VSMCs with LR-12 treatment due to decreased mitogens including cytokines [26]. This response may demonstrate an effect of attenuated inflammation to decrease proliferation of VSMCs and attenuate plaque formation [27]. No current studies have shown a direct relation between p27^kip1^ expression, TREM-1 stimulation, and the effect of TREM-1 inhibition. Additionally, the effect of the expression of OSM on the expression of p27^kip1^ has also not been studied. The results of this study highlight a possible relationship between these factors and events, however, their association with cell proliferation and plaque formation and vulnerability warrant further research.

In addition to biomarkers involved in VSMC proliferation, the focus of this study was to investigate the association of pro- and anti-inflammatory cytokine oncostatin M (OSM) [28] with plaque vulnerability. Prior studies have implicated OSM as one of the initiating molecules in atherosclerosis given its nature as an IL-6 family interleukin and its roles in modulating immune cell recruitment, endothelial activation, and adhesion molecules such as ICAM [11,29]. A decreased OSM expression in LR12-treated RAPA compared to its left counterpart may be due to attenuated inflammation by TREM-1 inhibition [28]. However, an increased OSM in LR-12 treated-peptide compared to scrambled peptide-treated RAPA raises concern and needs further investigation. A possible reason may be the presence of different subsets of T cells in atherosclerotic plaques and an increased secretion of OSM from these lymphocytes [30–32] and the presence of different subsets of T lymphocytes in treated and untreated arteries warrant investigation. Another reason may be the presence of adipocytes present in the ECM in collected samples in the RAPA [33]. The protein expression showed an increased immunopositivity for OSM in scrambled peptide-treated RAPA compared to LR12-treated RAPA and LAPA. An increased protein expression while decreased mRNA expression in scrambled peptide treated RAPA compared to LAPA and LR12 treated RAPA may be due to epigenetic influence, post-transcriptional and post-translational modification, or the changing microbiota/dysbiosis in plaque [34–36] and these aspects warrant further research.

To further elucidate whether these changes were occurring as a tissue-level response, we investigated the expression levels of OSMR, the receptor for OSM. The results revealed similar findings for fold change in mRNA expression as of OSM expression with the only difference that fold change in mRNA expression in LR-12 treated peptide was significantly decreased compared to RAPA treated with scrambled peptide. This may be due to a systemic immune response or due to TREM-1 inhibition by LR12, or the effects of high-fat high-cholesterol diet fed to these swine [9,37]. Although previous studies have demonstrated that the proinflammatory factor tumor necrosis factor-alpha (TNF-α) regulates TREM-1 while OSM regulates TNF-α-mediated inflammation, currently there are no reports suggesting a relationship between OSM and TREM-1 [11]. Thus, the direct regulatory mechanisms between OSM and TREM-1 warrant more studies. Additionally, it should also be noted that OSM is both an anti- and pro-inflammatory cytokine [28] and its pro- and anti-inflammatory function with various phases of plaque formation is not well understood. The differences between the gene and protein expression may also be due to the limited number of samples and increasing the sample size might be beneficial to establish a better relationship. mTOR plays a significant role in the pathogenesis of atherosclerosis [38]. mTOR, the molecular target of rapamycin, is implicated as a downstream factor of the proinflammatory pathway of OSM [39]. mTOR has been shown to regulate protein synthesis in response to amino acids, such as the PI3K pathway. Rapamycin has been shown to inhibit VSMC proliferation and promote differentiation, which may indicate a role for mTOR in the proliferative pathway [20]. An increased immunopositivity for mTOR in scrambled peptide-treated tissues compared to LR12-treated tissues suggests the pro-inflammatory effect of OSM and the potential of OSM-mTOR- p27^kip1^ axis to promote plaque development and vulnerability. This notion is supported by the fact that inflammatory signaling involving mTOR plays a critical role in atherosclerosis and inhibiting mTOR is therapeutically beneficial [40]. The role of mTOR in atherosclerosis is supported by the increased mRNA expression in RAPA treated with LR-12 and scrambled peptide compared to their counterpart. However, the effect of attenuating inflammation by LR-12 is reflected by attenuated mTOR in RAPA treated with LR-12 compared to scrambled peptide. Taken together, the results of this study suggest that intimal injury is associated with increased OSM, mTOR, and p27^kip1^ expression and investigating this axis in plaque formation to attenuate vulnerability will be worth investigating.

## Conclusion

While the complications and long-term effects of atherosclerotic plaque formation have been studied, the exact underlying mechanisms and pathways regarding plaque stability and vulnerability are still being uncovered. The results of this study delineate a potential association between OSM, mTOR, and p27^kip1^ expression and VSMC proliferation contributing to plaque size and vulnerability. These results should be further confirmed with increased sample size and in-vitro studies to establish the relationship between OSM-mediated mTOR activation, and attenuated p27^kip1^ expression in association with cell proliferation to correlate it with plaque stabilization using these mediators as potential therapeutic targets.

### Limitations of the study

While we performed a robust comparison of atherosclerotic lesions between microswine and contralateral arteries, our study faced limitations. One of the limitations could be the sample size of 7 tissues in each group. Another limitation is of slight variation in the results of gene markers analyzed by RT-PCR and IHC. This could be due to the nonavailability of the swine-specific antibodies. Western blot analysis for protein expression using swine-specific antibodies will be helpful to correlate the RT-PCR and IHC findings. The results of OSM-induced mTOR-mediated cell proliferation with attenuation of p27^kip1^ along with the effect of TREM-1 stimulation and inhibition need further investigation to support the findings using in-vitro studies. We are working on these aspects in our future experiments.

## Figures and Tables

**Table 1:** Nucleotide sequence of forward and reverse primers used in RT-PCR for mRNA transcript analysis of various genes.

Gene name	Forward primer	Reverse primer
OSM	5’-GAAGGGTTGGGTACCATGTAA-3’	5’-GTCTCTATGTCCGCAGAGAATC-3’
OSMR	5’-AACTGGCTCCTTCAGACAAC-3’	5’-GGCTGGGATTCAGTGGAATAA-3’
Ki-67	5’-GAAAGAGTGGCAACCTGCCTTC-3’	5’-GCACCAAGTTTTACTACATCTGCC-3’
α-SMA	5’-CCAGAGCAATCAGGGACC-3’	5’-CAATGGACGGGAAAACAGCC-3’
18S	5’-CCCACGGAATCGAGAAAGAG-3’	5’-TTGACGGAAGGGCACCA-3’

OSM- oncostatin M; OSMR-oncostatin M receptor; α-SMA- alpha-smooth muscle actin; Ki67- proliferation marker.
